# Novel Scanning Characterization Approaches for the Accurate Understanding and Successful Treatment of Oral and Maxillofacial Pathologies

**DOI:** 10.1155/2020/6545823

**Published:** 2020-02-19

**Authors:** Lavinia Cosmina Ardelean, Laura Cristina Rusu, Stefan G. Stanciu, Juan M. Bueno

**Affiliations:** ^1^Department of Technology of Materials and Devices in Dental Medicine, “Victor Babes” University of Medicine and Pharmacy from Timisoara, Timisoara, Romania; ^2^Department of Oral Pathology, “Victor Babes” University of Medicine and Pharmacy from Timisoara, Faculty of Dental Medicine, Timisoara, Romania; ^3^Center for Microscopy-Microanalysis and Information Processing, University Politehnica of Bucharest, Bucharest, Romania; ^4^Laboratorio de Optica, Universidad de Murcia, Murcia, Spain

Understanding in detail the modifications that occur in biological tissues during the progression of oral and maxillofacial pathologies requires the use of complementary scanning microscopy techniques. Optical imaging modalities such as multiphoton microscopy (MPM), Coherent Anti-Stokes Raman Scattering Microscopy (CARS), Reflectance Confocal Laser Scanning Microscopy (RCLSM), or optical coherence tomography (OCT) can thus be of great help for improving diagnosis, as they can visualize morphological features and provide information on biochemical modifications specific to various health states. Further on, these techniques can be used in tandem with other complex imaging tools, including Micro-CT, Scanning Electron Microscopy (SEM), or Atomic Force Microscopy (AFM), to shed light over the interactions that take place between soft tissues and advanced materials that are used in various therapeutic approaches, such as micro- and nanostructured polymers, ceramics, and metallic materials. These techniques have applications in diagnostics, theranostics, restorative and regenerative medicine, prosthetics, and other relevant biomedical tasks. Furthermore, to achieve a better understanding of oral and maxillofacial pathologies, these characterization techniques can be coupled with 3D scanning (e.g., intraoral scanning) and 3D printing technologies that are widely used in the dental area, because of their immense benefits. For example, scanning physical models into digital 3D computer-aided (CAD) files to be used in designing and additive manufacturing (CAM) of various prosthetic pieces, and aiding dentists with easier treatment planning, improved communication with laboratories and reduced operative and treatment time. The purpose of this special issue is to present recent progress made in scanning-based tissue imaging, material design and synthesis, and tissue-material interactions, which are relevant with respect to the accurate understanding and successful treatment of oral and maxillofacial pathologies. A brief summary of published papers is provided below.

In the paper of Paul Rotar et al., the accuracy of intraoral scanners used in the dental office, namely, two intraoral scanners Planmeca PlanScan (E4D Technologies, LLC, Richardson, TX, USA) and Omnicam CEREC (Sirona, Bensheim, Germany) as well as a high-resolution desktop scanner, D700, (3Shape, Copenhagen, Denmark), was evaluated. Trueness values were obtained by superimposing the STL files from the test groups with the STL file from the reference scan. Overlapping the STL files within each group generated the precision values. For each set of scans, the mean and standard deviation values were calculated. Statistical analysis was performed using the Kolmogorov-Smirnov test to assess data distribution. Overall trueness and precision of the scanners were analyzed and compared, and the statistical significance was calculated using the paired *t*-test. The results showed that accuracy deviations of the analyzed scanners were consistent and with no major differences between them.

Michaela Relucenti et al. presented a novel scanning characterization approach, the BSE 3D image analysis, to study the pathological erosion on the surface of human incus bone involved by cholesteatoma, in order to assess the eventual osteoclastic resorptive action. BSE 3D images of resorption pits from osteoporotic human femur neck with that of the incus were compared. Surface parameters were calculated by the software Hitachi Mountains Map© from BSE 3D-reconstructed images; results were then statistically analyzed by SPSS statistical software. The conclusion was that no significant differences exist between the two groups. This quantitative approach implements the morphological characterization, allowing to state that surface erosion of the incus is due to osteoclasts' action. Novel scanning characterization approaches used allowed the 3D imaging of incus bone erosion and its quantitative measurement, opening a new era of quantitative SEM morphology.

Francesco Guido Mangano et al. aimed to present a digital method that combines intraoral and face scanning for the CAD/CAM fabrication of implant-supported bars for maxillary overdentures. 15 patients were rehabilitated with a maxillary overdenture supported by a CAD/CAM polyether-ether-ketone (PEEK) implant-supported bar. The outcomes of the study were the passive fit/adaptation of the bar, the 1-year implant survival, and the success rates of the implant-supported overdentures. The 1-year success rate was of 80% for the implant-supported overdenture leading to the conclusion that the combination of intraoral and face scans allowed to successfully restore fully edentulous patients.

In conclusion, the objectives of the special issue have been reached, several aspects of important interest have been addressed, and the proposed contributions exhibit promising results that outperform existing studies.

